# Supplementary figures and images for: Eicosapentaenoic Acid Regulates Inflammatory Pathways through Modulation of Transcripts and miRNA in Adipose Tissue of Obese Mice
Source: Biomolecules. 2020 Sep 7;10(9):1292. doi: 10.3390/biom10091292 (PMC7564513; doi:10.3390/biom10091292)

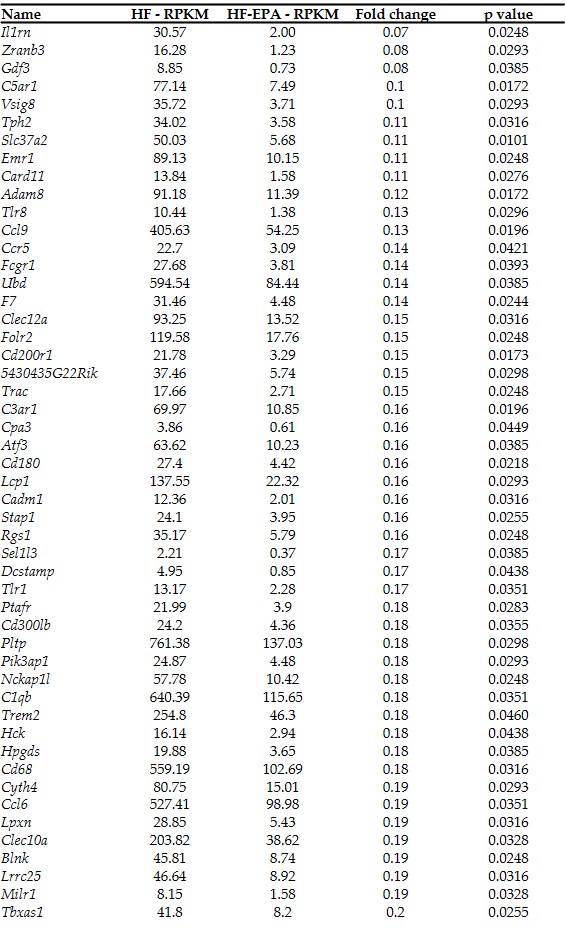

Supplement: Supplementary file 1 [file biomolecules-10-01292-s001.zip › Sup table 1.1.jpg]

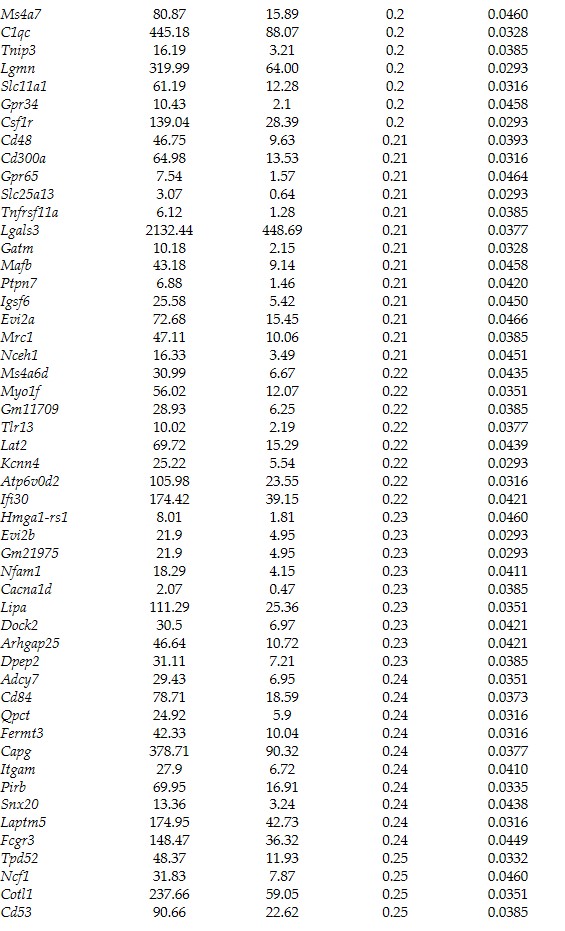

Supplement: Supplementary file 1 [file biomolecules-10-01292-s001.zip › Sup table 1.2.jpg]

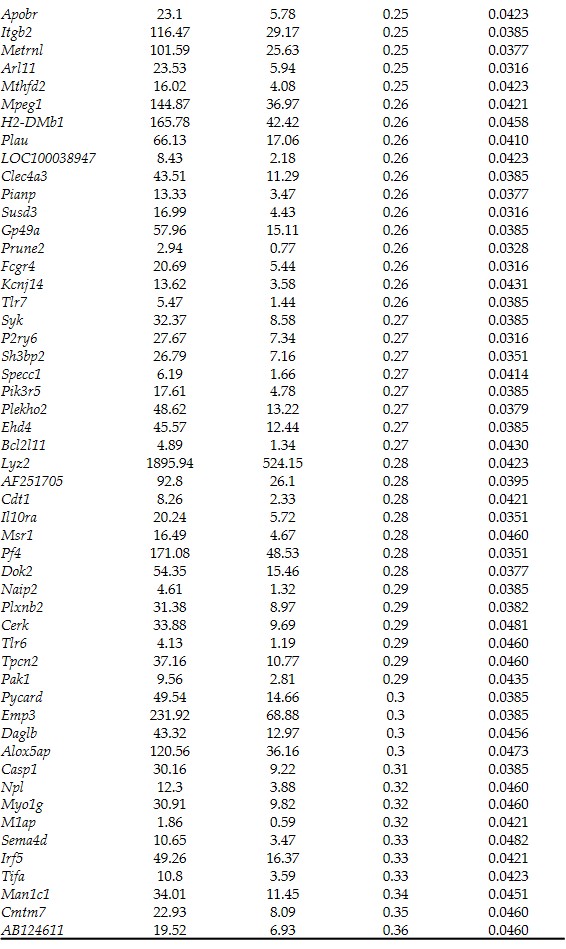

Supplement: Supplementary file 1 [file biomolecules-10-01292-s001.zip › Sup table 1.3.jpg]

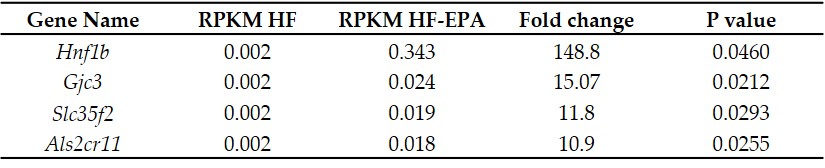

Supplement: Supplementary file 1 [file biomolecules-10-01292-s001.zip › Sup table 2.jpg]

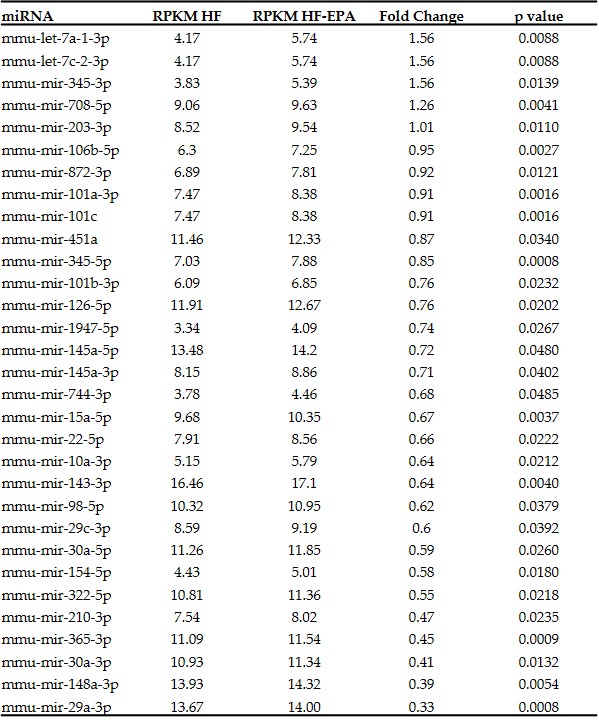

Supplement: Supplementary file 1 [file biomolecules-10-01292-s001.zip › Sup table 3.jpg]

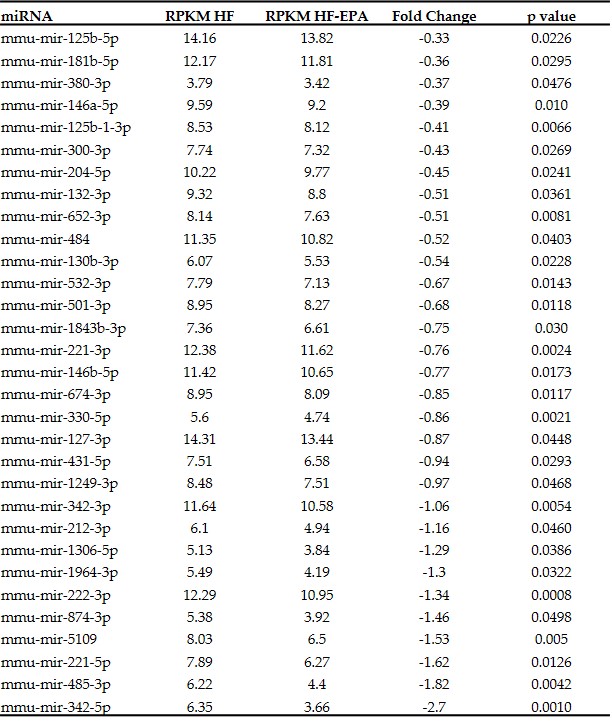

Supplement: Supplementary file 1 [file biomolecules-10-01292-s001.zip › Sup table 4.jpg]
